# The fission yeast MTREC complex targets CUTs and unspliced pre-mRNAs to the nuclear exosome

**DOI:** 10.1038/ncomms8050

**Published:** 2015-05-20

**Authors:** Yang Zhou, Jianguo Zhu, Géza Schermann, Corina Ohle, Katja Bendrin, Rie Sugioka-Sugiyama, Tomoyasu Sugiyama, Tamás Fischer

**Affiliations:** 1 Biochemistry Center (BZH), Heidelberg University, Heidelberg 69120, Germany; 2 Faculty of Life and Environmental Sciences, University of Tsukuba, Tsukuba, Ibaraki 305-8577, Japan; 3 Life Science Center of Tsukuba Advanced Research Alliance, University of Tsukuba, Tsukuba, Ibaraki 305-8577, Japan

## Abstract

Cryptic unstable transcripts (CUTs) are rapidly degraded by the nuclear exosome. However, the mechanism by which they are recognized and targeted to the exosome is not fully understood. Here we report that the MTREC complex, which has recently been shown to promote degradation of meiotic mRNAs and regulatory ncRNAs, is also the major nuclear exosome targeting complex for CUTs and unspliced pre-mRNAs in *Schizosaccharomyces pombe*. The MTREC complex specifically binds to CUTs, meiotic mRNAs and unspliced pre-mRNA transcripts and targets these RNAs for degradation by the nuclear exosome, while the TRAMP complex has only a minor role in this process. The MTREC complex physically interacts with the nuclear exosome and with various RNA-binding and RNA-processing complexes, coupling RNA processing to the RNA degradation machinery. Our study reveals the central role of the evolutionarily conserved MTREC complex in RNA quality control, and in the recognition and elimination of CUTs.

## Abstract (web summary)

The evolutionarily conserved MTREC complex promotes degradation of meiotic mRNAs and regulatory ncRNAs. Here the authors show that MTREC also targets cryptic unstable transcripts and unspliced pre-mRNAs for degradation by the nuclear exosome, while the TRAMP complex has only a minor role in this process.

## Graphical abstract

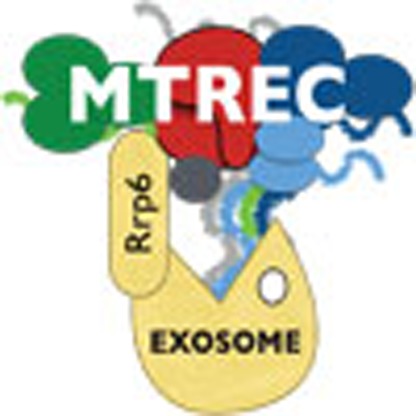

The recent development of new techniques for transcriptome analyses has uncovered another layer of complexity in the eukaryotic transcriptome. Studies using these techniques have revealed that genomic regions previously thought to be silent, such as heterochromatic regions, intergenic regions and antisense (AS) portions of the genome, are actually transcriptionally active and produce a significant amount of non-protein coding RNA (ncRNA) transcripts^1^,^2^. In yeast, a large portion of these ncRNAs, termed cryptic unstable transcripts (CUTs), is quickly degraded by the nuclear exosome. The exosome is the major 3′ to 5′ RNA degradation machinery that plays a key role in RNA metabolism, RNA processing and RNA surveillance, and it is essential for the viability of eukaryotic cells. This evolutionarily conserved multi-subunit complex is formed by a 10 subunit core complex and some compartment-specific subunits^3^,^4^. One such subunit is Rrp6, which is exclusively associated with the nuclear form of the exosome, and is required for the rapid nuclear degradation of CUTs. In fact, CUTs were classically defined as transcripts stabilized by the deletion of *rrp6* (refs 5, 6). Unlike the core exosome, Rrp6 is not essential for cell viability in yeast, although *rrp6Δ* shows a severe growth defect.

The activity and target specificity of the exosome in the yeast *Saccharomyces cerevisiae (S. cerevisiae)* is regulated by compartment-specific cofactors, such as the nuclear TRAMP complex^5^,^7^,^8^ or the cytoplasmic Ski-complex^9^. Both of these complexes contain a related DExH-box RNA helicase subunit, Mtr4 and Ski2, respectively. The helicase activity of these complexes plays a role in the unwinding of structured RNA templates, and channels them to the exosome^10^. The TRAMP complex consists of a non-canonical poly(A) polymerase Trf4 or Trf5, a Zn-knuckle protein Air1 or Air2, and the RNA helicase Mtr4 subunits. This complex adds short oligo(A) tails to CUTs and feeds these RNAs through the Rrp6 subunit to the core exosome^11^,^12^,^13^,^14^,^15^. In addition to the recognition and degradation of CUTs, the TRAMP complex and Rrp6 also play an essential role in the processing of ribosomal RNA (rRNA), transfer RNAs and small nuclear and nucleolar RNAs^16^. Although the TRAMP complex is evolutionarily conserved, the function of the mammalian TRAMP complex seems to be more restricted to rRNA biogenesis^17^. However, the human orthologue of the RNA helicase Mtr4 (hMTR4) is also part of the trimeric nuclear exosome targeting (NEXT) complex together with the Zn-finger protein, ZCCHC8, and an RNA-binding protein, RBM7. The NEXT complex physically interacts with the cap-binding complex (CBC) and the ARS2 protein (CBCA complex), forming the CBC–NEXT (CBCN) complex^18^,^19^. The NEXT and CBCN complexes are responsible for the recognition of the promoter upstream transcripts (PROMPTs) in mammalian cells and for targeting them to the nuclear exosome^17^.

In the fission yeast *Schizosaccharomyces pombe* (*S. pombe*), the nuclear exosome is also involved in the degradation of a group of meiotic messenger RNAs (mRNAs) during vegetative growth. The targeting of these mRNAs to the nuclear exosome is independent of the TRAMP complex^20^,^21^, but requires the RNA-binding protein Mmi1, which can recognize a specific sequence motif (determinant of selective removal or DSR-sequence) on meiotic mRNAs^22^,^23^,^24^. Several additional proteins were reported to participate in this specific meiotic mRNA elimination system, including Red1 (RNA elimination defective 1)^25^, Pab2 (refs 21, 26), Red5 (ref. 27) and Iss10 (ref. 28). Interestingly, *S. pombe* has a second Mtr4 homologue protein, Mtl1 (Mtr4-like protein 1). Mtl1 was recently shown to interact with Red1, Pab2, Red5, Iss10, Mmi1 and several additional nuclear proteins^29^,^30^, some of which are related to the subunits of the human CBCN complex. This complex, also called MTREC (Mtl1–Red1 core) or NURS (nuclear RNA silencing) complex, is not only responsible for the degradation of meiotic mRNAs and ncRNAs, but also plays a role in the assembly of heterochromatic islands at meiotic genes^29^,^30^,^31^,^32^,^33^. Mtl1 also forms a separate complex with Nrl1 and Ctr1 and interacts with the splicing machinery, and can target intron-containing precursor telomerase RNA and cryptic introns to facilitate splicing and the production of short interfering RNAs at these loci^29^.

To further understand the role of this complex in RNA surveillance, we analysed the transcriptome of *S. pombe* cells carrying mutations in subunits of the MTREC complex. In addition to increased meiotic mRNA levels, these mutants also show strong, genome-wide accumulations of CUTs and unspliced pre-mRNA transcripts, at a comparable level to the nuclear exosome subunit, *rrp6* deletion. Conversely, deletion of the TRAMP complex subunit *cid14* shows only slight accumulation of these transcripts, indicating that in *S. pombe* the TRAMP complex has only a minor role in this process. RNA immunoprecipitation (RIP) experiments reveal that the MTREC complex is specifically recruited to CUTs and meiotic mRNAs. Our findings establish the MTREC complex as a central component of the eukaryotic RNA surveillance machinery through its role in the recognition and delivery of CUTs and unspliced/mis-spliced pre-mRNAs to the nuclear exosome.

## Results

### MTREC forms a large complex that interacts with the exosome

To understand more about the biochemical composition of the MTREC complex, we tagged the genomic copy of Mtl1 and Red1 and some of the interacting proteins, including Red5, Ars2 and Cbc1. We used tandem affinity tags and purified them under physiological salt concentrations. Furthermore, we also carried out these purifications in deletion alleles of various subunits to map interactions within the complex. The samples were treated with Benzonase, a highly efficient nuclease, to eliminate RNA- or DNA-mediated interactions. Co-purifying proteins were determined by matrix-assisted laser desorption/ionization–time of flight analysis of single protein bands and the liquid chromatography–tandem mass spectrometry analysis of the whole protein purification mix. We used label-free quantification (intensity-based absolute quantification, iBAQ)^34^ to determine the relative abundance of co-purified proteins compared with the bait protein.

**Figure 1:**
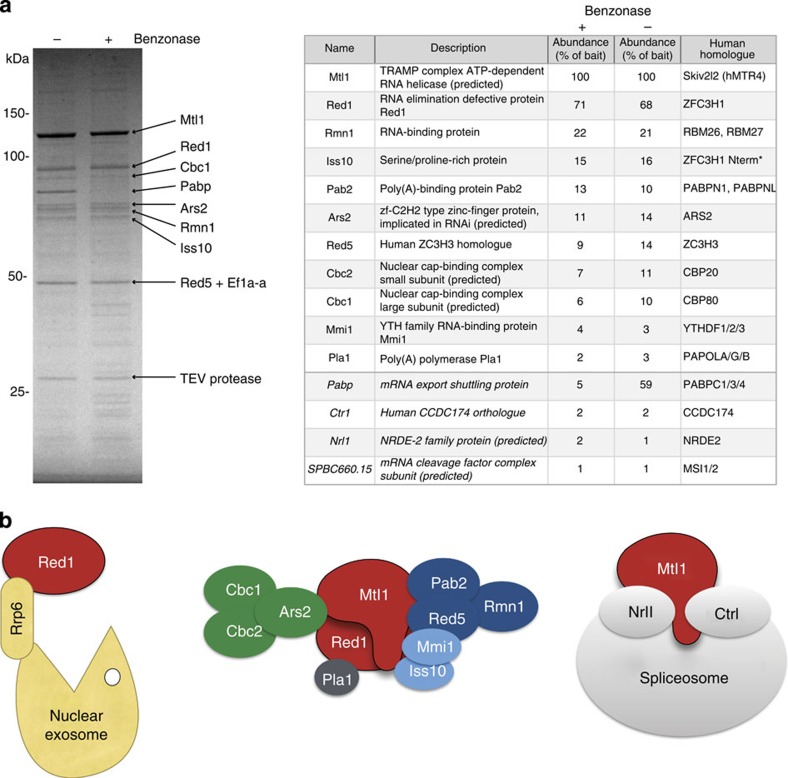
MTREC complex consists of five submodules and interacts with the nuclear exosome and the spliceosome. (**a**) The Coomassie blue stained SDS polyacrylamide gel shows the tandem affinity-purified MTREC complex, using Mtl1 as bait, in the presence (+) or absence (−) of Benzonase. Proteins identified by mass spectrometry of excised bands are indicated by arrows. The table displays the results of the LC-MS/MS analysis of the purified total protein fractions showing the abundance of the identified proteins normalized to the bait, as a percentage. Italicized proteins weakly interact with the MTREC complex, but are not part of the complex. The names of the closest human homologue proteins are indicated in the last column. * Iss10 shows high homology to the N-terminal region of ZFC3H1. The purifications were performed in two biological replicates. (**b**) Schematic representations of the identified complexes and submodules. The different colours correspond to the submodule organization of the MTREC complex, as also shown in Table 1. LC-MS/MS, liquid chromatography–tandem mass spectrometry.

These analyses confirmed several previously detected interactions^28^,^29^,^30^, and also significantly extended our understanding of this large, multi-subunit complex. We identified nine proteins that bind to both Mtl1 and Red1, and the binding was insensitive to nuclease treatment (Fig.1a). In addition to the previously identified Mtl1–Red1 core module, we identified four submodules: Red5–Pab2–Rmn1, Ars2–Cbc1–Cbc2, Iss10–Mmi1 and the more loosely associated canonical poly(A) polymerase Pla1 (Table 1; Fig. 1b; Supplementary Fig. 1a,b; Supplementary Data 1, 2). Evidence for the existence of these submodules was collected from the following observations. Deletion of *red1* destroyed the complex as a whole, but the Red5–Pab2–Rmn1 trimeric complex could still be isolated by purifying the Red5 subunit. Furthermore, deletion of *pab2* dissociated Red5 and Rmn1 from the core module, but the rest of the complex remained intact, although with impaired stability. Deletion of *rmn1* also negatively affected the overall stability of the MTREC complex, but did not interrupt specific interactions, indicating a more peripheral role for Rmn1. The trimeric Cbc1–Cbc2–Ars2 complex, which is the *S. pombe* equivalent of the human CBCA complex, could be isolated using Ars2 or Cbc1 as baits. These purifications also revealed that under steady-state conditions, only a small fraction of the Cbc1–Cbc2–Ars2 complex is stably associated with the MTREC complex. The Mmi1 subunit can be co-purified with the Pab2–Red5–Rmn1 submodule (see Red5–FTP in *red1Δ*), but also with the core-subunit Mtl1 alone (see Mtl1–FTP in *red1Δ* or Nrl1–FTP/Ctr1–FTP), indicating that Mmi1 has multiple interaction sites within the complex. Consistent with previous studies^28^, Mmi1 binding to the complex is strongly impaired by the deletion of the Iss10 subunit, while other subunits were not affected in *iss10Δ*.

Using Red5 as bait, we recovered not only the core module and the Red5–Pab2–Rmn1 submodule, but also Cbc1–Cbc2–Ars2 and Iss10–Mmi1. Furthermore, we used a split-tag approach to purify Mtl1 and, as a second purification step, Iss10, Cbc1 or Pab2. All of these purifications efficiently recovered all 11 subunits of the complex (Table 1; Supplementary Fig. 1a,b; Supplementary Data 1, 2), demonstrating that the Mtl1–Red1 core module can associate simultaneously with all submodules, forming a large, 11 subunit complex. However, the different stoichiometry of the submodules indicates that the MTREC complex might exist in various forms, and the different submodules can dynamically associate/dissociate with/from the Mtl1–Red1 core module.

The MTREC complex also interacts with the nuclear exosome, co-purifying a small (<1% of the bait protein), but significant amount of all 12 nuclear exosome subunits, including Rrp6 and Mpp6 (Supplementary Fig. 1a,b; Supplementary Data 1, 2). However, purification of the Red1 subunit yielded a particularly efficient isolation of the nuclear exosome (>20% of the bait), indicating that the interaction between the nuclear exosome and the MTREC complex is likely to be mediated by Red1. The exosome subunit Rrp6 was essential for this interaction, since in the *rrp6Δ* background, exosome subunits did not co-purify with the MTREC complex (Table 1; Fig. 1b; Supplementary Fig. 1a,b; Supplementary Data 1, 2).

In addition to the poly(A) polymerase, Pla1, the mRNA cleavage factor complex subunit, SPBC660.15 (homologue of *S. cerevisiae* Hrp1), was also found consistently in the purifications (Fig. 1a; Supplementary Data 1, 2), supporting the suggested interaction of the MTREC complex with cleavage and polyadenylation factors^25^,^29^,^30^. As previously reported, Mtl1 also forms a trimeric complex with Nrl1 and Ctr1 and interacts with the spliceosome independently from Red1 and other MTREC subunits^29^ (Table 1; Fig. 1b; Supplementary Fig. 1a,b; Supplementary Data 1, 2).

### MTREC targets CUTs to the nuclear exosome for degradation

**Figure 2:**
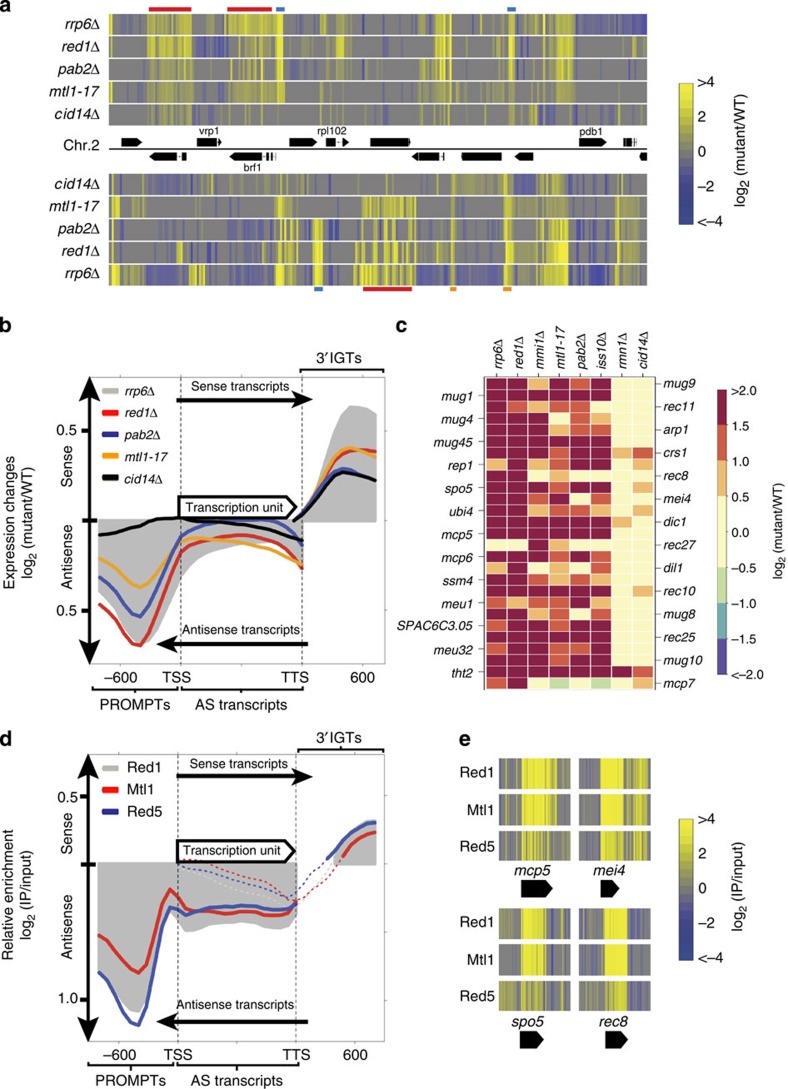
MTREC complex is the major nuclear exosome targeting factor in *S. pombe*. (**a**) Heat map of a portion of the *S. pombe* genome showing relative levels of transcripts from the forward (upper panel) and reverse (lower panel) strand in the indicated strains compared to WT. The middle panel shows genes and their directions. Colours represent up- or down-regulation of transcripts on a log_2_ scale. Red, blue and orange bars highlight examples of AS transcripts, PROMPTs and 3′IGTs, respectively. (**b**) Composite plot of relative RNA levels in the indicated mutant strains, compared to WT. Each gene was divided into 15 parts (bins) and the average fold-change in RNA level (compared with WT) in the sense and AS direction was determined for each bin. In addition, 800 bp from the flanking 5′ regions were included in the AS expression analysis, and 800 bp from the flanking 3′ regions were included in the sense expression analysis. In the resulting matrix, each row represents a gene and each column represents the position in the gene region (flanking regions and bins 1–15). The average of the log_2_ values (geometric average) was determined for each column and plotted on a log_2_ scale separately for sense (upper plot) and AS (lower plot with mirrored axis) transcripts. Only positive values are shown on the plots. The grey shaded area represents the average expression changes in the *rrp6Δ* strain and coloured lines in the indicated strains. (**c**) Expression changes of genes in the Mmi1-regulon in the indicated mutant strains. Colours represent the relative mRNA level of the indicated gene compared to WT. (**d**) Composite plot of the RIP experiments using tandem-affinity-tagged versions of the indicated proteins. The composite plot was plotted as described for panel b with the exception that negative values were also plotted (dotted line). Values represent the over-enrichment (solid line) or under-enrichment (dotted line) of the transcripts in the IP fraction compared to input. (**e**) Heat maps showing sense transcripts of the indicated meiotic genes. Colours represent enrichment in the IP fraction compared to input.

To obtain new insights into the role of the MTREC complex in RNA processing, we carried out expression profiling experiments with a temperature-sensitive allele of *mtl1* (*mtl1-17*) or deletion alleles (*red1Δ*, *iss10Δ*, *mmi1Δ*, *rmn1Δ* and *pab2Δ*) of the complex. We also included deletion alleles of the nuclear exosome subunit *rrp6*, and the TRAMP complex subunit, *cid14*, in our analysis. We used high-resolution tiling microarrays with strand-specific probes to monitor changes in RNA levels compared with a wild-type (WT) strain. As previously reported, deletion of *rrp6* leads to strong accumulation of CUTs and a small group of mRNAs that are mostly involved in meiosis^16^,^23^,^25^,^29^,^30^,^35^. CUTs in *S. pombe* can be divided into three major categories as follows: (1) short PROMPTs, initiating from the vicinity of the transcription start site (TSS) in the AS direction of the associated genes; (2) AS transcripts within gene regions; and (3) short 3′-intergenic transcripts (3′IGTs) downstream of the transcription termination site (TTS), which corresponds to the sense direction of the associated genes (Fig. 2a,b; Supplementary Fig. 2a,b).

Surprisingly, deletion of the *S. pombe* Trf4/5 orthologue, *cid14*, showed only a minor effect on CUTs at a genome-wide level (Fig. 2a,b). While AS transcripts and 3′IGTs showed minor accumulation in the *cid14Δ* strain, PROMPTs and meiotic mRNAs were only negligibly affected (Fig. 2b,c). This result suggests that the TRAMP complex is mostly dispensable for the recognition and degradation of CUTs in *S. pombe*. In contrast, deletion or mutation alleles of the MTREC complex lead to significant accumulation of all types of CUTs and also meiotic mRNAs. This effect is comparable to the level of CUT accumulation in the nuclear exosome subunit *rrp6* deletion (Fig. 2a,b). Strand-specific RNA-sequencing (RNA-seq) of *rrp6Δ*, *red1Δ* and *mtl1-17* strains also confirmed the microarray results (Supplementary Fig. 2b), and showed elevated PROMPTs, AS RNAs and 3′IGTs in these mutants compared with the WT strain.

A closer look at the expression profiles also reveals remarkable differences between the effects of the different subunit deletions (Fig. 2b,c; Supplementary Fig. 2a,b). While *red1Δ* equally affects all subtypes of CUTs and meiotic mRNAs, *pab2Δ* mostly accumulates PROMPTs, but AS transcripts are only marginally affected. Deletion of *iss10* or *mmi1* only affects meiotic mRNAs, while *rmn1Δ* shows only a moderate accumulation of PROMPTs. These results, together with previous reports^29^,^30^, suggest that the submodules of the complex might also represent functional modules, responsible for the targeting of different RNA subclasses.

### MTREC is specifically recruited to CUTs and meiotic mRNAs

The majority of the MTREC subunits possess potential RNA-binding motifs, suggesting that the complex might directly bind to various RNA transcripts. To further understand the role of this complex in the recognition and degradation of CUTs, we carried out RIP experiments to determine the RNA-binding profile of the MTREC complex. We used tandem-affinity-tagged versions of Red1, Mtl1 or Red5 subunits to purify the complex and analyse the co-purifying RNA fraction. These purifications yielded a significant amount of RNA, which we reverse transcribed, labelled and hybridized to tiling microarrays against RNA isolated from the input of the corresponding strain. These experiments revealed that the immunoprecipitated (IPed) RNA fraction is strongly enriched for CUTs, such as PROMPTs, AS transcripts and, to a lesser extent, 3′IGTs (Fig. 2d). Meiotic mRNAs regulated by the nuclear exosome were also highly enriched in the IP fraction (Fig. 2e), while other mRNAs were generally under-represented. Under our experimental conditions, each of the three bait proteins purified the entire MTREC complex, and accordingly, they showed very similar RNA-binding profiles to each other. We also carried out strand-specific reverse transcription PCR (RT–PCR) reactions to amplify selected CUTs and the *mei4* meiotic mRNA and confirmed the strong enrichment of these transcripts in the IP fraction compared with the input (Supplementary Fig. 2c). Overall, our experiments show that the MTREC complex is specifically recruited to CUTs and meiotic mRNAs, and it plays a key role in their degradation by the nuclear exosome.

### CUTs and meiotic mRNAs have long poly(A) tails

**Figure 3:**
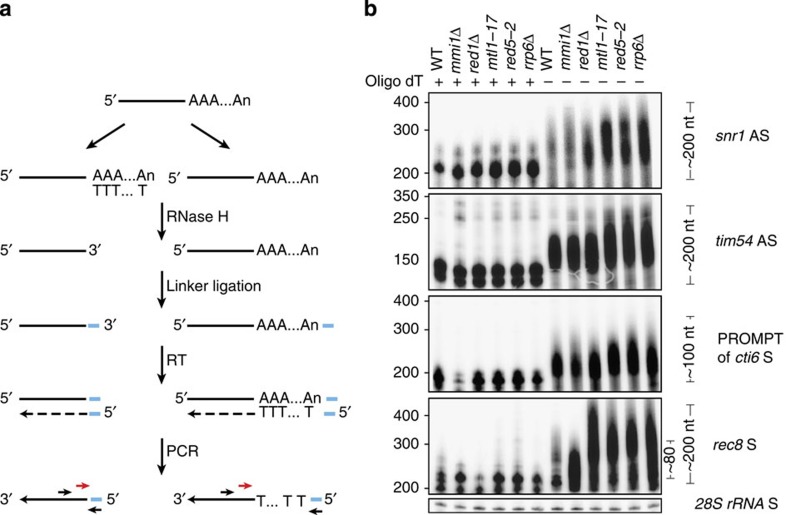
CUTs and meiotic mRNAs have long poly(A) tails. (**a**) Schematic representation of RNA ligation-coupled RT–PCR experiments. The blue box represents the 3′ linker, the small black arrows represent the gene-specific and the linker-specific primers to amplify the 3′ end of the transcripts, and the red arrows represent the ^32^P-labelled primer. (**b**) RNA ligation-coupled RT–PCR assay to assess the poly(A) tail length of the indicated transcripts in WT and mutant cells. Total RNA samples isolated from the indicated strains were treated with RNase H in the presence (+) or absence (−) of oligo(dT) DNA fragments. The ^32^P signals of the labelled PCR products were visualized after DNA electrophoresis. DNA marker is indicated on the left. 28S rRNA was used as a loading control.

The poly(A) polymerase activity of the TRAMP complex adds a short oligo(A) tail to its substrate RNAs^13^,^14^. This short oligo(A) tail is important for the activation of the exosome, probably by establishing an unstructured 3′ end to initiate the exonucleotic degradation^36^. Since the MTREC complex also interacts with the canonical poly(A) polymerase, Pla1 (refs 25, 29, 30), and other cleavage factors (Fig. 1a), and the poly(A) tail on meiotic mRNAs is a prerequisite for their efficient degradation^21^,^23^,^25^, we wondered whether CUTs are also polyadenylated in *S. pombe*. We treated total RNA of WT or MTREC mutant strains with RNase H in the presence or absence of an oligo(dT) DNA oligomer. The RNase H activity cleaves the poly(A) tails of the RNA transcripts only in the presence of the complementary oligo(dT) DNA. After ligating linkers to the 3′ end of the RNase H-treated transcripts, we generated a complementary DNA (cDNA) library and amplified the 3′ ends of selected transcripts^37^ (Fig. 3a,b; Supplementary Fig. 3). We analysed the length of the poly(A) tails of two cryptic AS transcripts, the PROMPT of the *cti6* gene and used the *rec8* meiotic mRNA as a control^23^,^25^,^27^ (Fig. 3b). Our analysis showed that these transcripts are all polyadenylated both in WT and in mutant cells, and the poly(A) tail can be up to 200 nucleotides in length. Mutations in the MTREC complex, or deletion of *rrp6*, did not significantly change the length of the poly(A) tails of these CUTs, with the exception of the *pla1-ts37* mutant, which showed decreased poly(A) tail length, but also caused strong alternative cleavage site usage at the analysed AS transcripts (these bands are also visible in the RNA fractions treated with oligo(dT)) (Supplementary Fig. 3). The poly(A) tail of *rec8* was significantly shorter in the *mmi1Δ* strain, reaching only about 80 nucleotides in length, which is a typical poly(A) length for mRNAs in *S. pombe*. This observation is consistent with previous reports that Mmi1 promotes hyperadenylation of DSR-containing meiotic mRNAs and is required for their degradation by the nuclear exosome^21^,^23^,^25^, and shows that in the absence of Mmi1, DSR-containing transcripts are processed as normal mRNAs. Since the amount of *rec8* transcript is very low in WT cells, we could not reliably determine the length of the poly(A) tail in WT conditions. These results show that, in contrast to *S. cerevisiae*, CUTs have long poly(A) tails in *S. pombe*. Since the typical poly(A) tail length of mRNAs is about 50–80 nucleotides in fission yeast, CUTs and meiotic mRNAs are relatively hyperadenylated. This result is also consistent with the very high abundance of the poly(A)-binding protein, Pabp, in the purification of the MTREC complex (Fig. 1a). The co-purification of Pabp was highly sensitive to nuclease treatment, suggesting that Pabp binding is mediated by RNAs with long poly(A) tails.

### Mtl1–Ctr1–Nrl1 targets unspliced pre-mRNAs to MTREC

**Figure 4:**
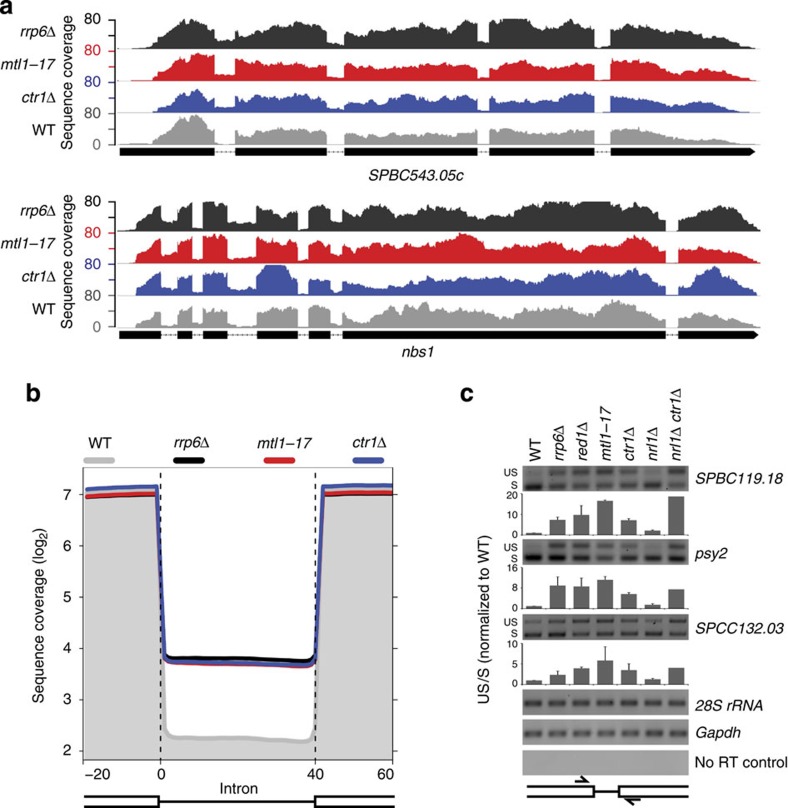
The Mtl1–Ctr1–Nrl1 complex targets unspliced pre-mRNA products for degradation. (**a**) Strand-specific RNA-seq read coverage of the indicated genes in WT and mutant strains. (**b**) Composite plot of RNA-seq read coverage including all introns in genes with detectable expression (3,935 introns). Each intron was divided into 40 parts (bins) and the average RNA sequence read coverage (only sense transcripts) was determined for each bin. In addition, the surrounding 20-base-pair exonic regions were also included. In the resulting matrix, each row represents an intron and each column represents the position in the intronic region (flanking exonic regions and bins 1–40). The average of the log_2_ values (geometric average) was determined for each column and plotted on a log_2_ scale. The grey shaded area represents the average RNA levels in the WT strain and coloured lines in the indicated mutant strains. (**c**) Strand-specific RT–PCR results of exon–exon junctions in WT and indicated mutant strains. Smaller PCR product (lower band) represents the spliced (S) transcripts, the longer product (upper band) represents the unspliced (US) transcripts in the indicated genes. The bar chart shows the ratio between unspliced and spliced products (US/S), normalized to the US/S ratio in the WT strain. Error bars indicate s.d. values. 28S rRNA and *GAPDH* were used to control equal RNA loading. A no RT control was performed for each locus, and showed no detectable products (the *psy2* no RT control is shown as an example). The RT–PCR reactions were performed in two biological replicates, with the exception of the *nrl1Δctr1Δ* mutant, where no replicate was performed.

Mtl1 also forms a separate complex with Ctr1 and Nrl1, which interacts with the spliceosome and was reported to function in cryptic splice site utilization at several genomic sites^29^. These findings prompted us to analyse the potential role of Mtl1 and the MTREC complex in the quality control of splicing. The role of the exosome in the degradation of unspliced pre-mRNA transcripts was previously documented^16^,^38^,^39^, but the molecular mechanism underlying how these transcripts are recognized by the exosome is not understood. We used RNA-seq technology as an alternative to microarray because, in general, microarray data do not give the necessary resolution to reliably detect the short intronic sequences typically present in *S. pombe*. We isolated total RNA from WT and mutant cells, depleted the rRNAs, and prepared directional RNA libraries for Illumina sequencing. Similar to previous reports, we detected significantly increased intronic reads in the exosome mutant *rrp6Δ* strain (Fig. 4a,b). Remarkably, we observed a very similar pattern in the *mtl1-17* mutant, as well as in the *ctr1Δ*, *red1Δ* and, to a slightly lesser extent, in the *nrl1Δ* strains (Fig. 4a,b; Supplementary Fig. 4a). Composite plots of all introns in the *S. pombe* genome show two to three times higher intronic reads in the mutants compared with WT. This increase in the intronic reads can arise from the elevated levels of the excised intronic sequences or higher amounts of unspliced or mis-spliced RNA transcripts. Closer analysis of the sequencing reads showed that these intronic reads mostly span exon–intron boundaries (Supplementary Fig. 4b), demonstrating that the increased intronic reads are the result of the elevated levels of unspliced or mis-spliced pre-mRNA transcripts. To further confirm this finding, we selected introns from three different genes and determined the levels of spliced and unspliced RNA in WT and mutant strains. We used strand-specific RT–PCR to amplify short sense RNA fragments spanning the splice site, resulting in a short PCR product from spliced RNAs, and a longer product from unspliced transcripts. Compared with the WT strain, the amount of unspliced products increased markedly in *rrp6Δ*, *red1Δ*, *mtl1-17*, *ctr1Δ*, *nrl1Δctr1Δ* strains and, to a lesser extent, in the *nrl1Δ* strain (Fig. 4c). Since the primers hybridized to the exonic sequences, the longer PCR products are produced exclusively from unspliced transcripts. These results confirm the accumulation of unspliced or mis-spliced pre-mRNA transcripts in these mutant strains.

Increased levels of unspliced pre-mRNA transcripts might point to impaired quality control and the inefficient degradation of unspliced/mis-spliced transcripts, or, alternatively, the splicing process itself might be affected in these mutants. If a mutation stabilizes unspliced pre-mRNAs, in addition to the elevated intronic RNA levels, exonic coverage would also be expected to show a similar increase. In contrast, in case of a splicing defect, the level of exonic sequence coverage would be unaffected or show a moderate decrease if the unspliced transcripts are less stable than the properly spliced transcripts. Since the absolute increase in the intronic reads is relatively low compared with the level of exonic reads, we carried out the analysis on introns representing the upper 25 percentile with the highest increase of intronic coverage normalized to the expression level of the surrounding exons in the *ctr1Δ* mutant strain (Supplementary Fig. 4c,d, see the figure legend and Methods section for a more detailed description of the analysis). This analysis revealed that in the *ctr1Δ* or *nrl1Δ* mutant strains, both the intronic and also the surrounding exonic sequence coverage showed similar increases, while the expression of genes without introns was unaffected (Supplementary Fig. 4c,d). This result strongly suggests that the elevated level of intronic reads in the mutant strains is the consequence of the inefficient degradation of unspliced or mis-spliced transcripts; however, a defect in splicing cannot be completely ruled out. Furthermore, the significant increase in the level of intronic reads is not only detected in mutants of *mtl1*, *nrl1* and *ctr1*, factors that tightly interact with the spliceosome, but also in *red1Δ* or *rrp6Δ* strains, even though these factors are not a part of the spliceosome and are unlikely to directly affect genome-wide splicing efficiency. Overall, our data strongly indicates that the Mtr1–Ctr1–Nrl1 complex is involved in the recognition and degradation of unspliced or mis-spliced RNA products through the MTREC complex and the nuclear exosome.

## Discussion

Widespread pervasive transcription in the eukaryotic genome produces a substantial amount of non-protein coding transcripts, but the majority of these ncRNAs are unstable and rapidly degraded by the RNA surveillance machinery. The nuclear exosome, a large complex with 3′ to 5′ exonuclease activity, is mostly responsible for the degradation of these ncRNAs. The substrate specificity of this very efficient RNA degradation machinery is tightly controlled by specific exosome targeting complexes that present substrate RNAs to the exosome for degradation. Such exosome targeting complexes include the well-characterized nuclear TRAMP complex, the cytoplasmic Ski complex or the human NEXT complex. The recently identified *S. pombe* MTREC complex was also shown to target meiotic mRNAs and regulatory ncRNAs to the nuclear exosome^29^,^30^. Our results further show that the MTREC complex is the major nuclear exosome targeting factor for CUTs and unspliced pre-mRNAs and it is required for the rapid degradation of these transcripts. The helicase subunit Mtl1 also interacts with the spliceosome and plays a key role in the quality control process during splicing by targeting unspliced or mis-spliced pre-mRNA transcripts to the exosome.

Interestingly, all of the exosome targeting complexes identified to date contain a conserved RNA helicase. On the basis of the available structural data for the Ski-complex and the TRAMP complex, it is likely that the helicase activity plays an essential role in the unwinding of the target RNAs and in feeding them directly into the barrel-like structure of the exosome^10^,^40^,^41^. Similarly, Mtl1, the helicase subunit of the MTREC complex, is likely to have a role in feeding the MTREC-bound RNAs into the nuclear exosome. Although Mtl1 is highly similar to Mtr4, the short N-terminal motif responsible for the Mtr4 interaction with the exosomal subunits Rrp6 and Rrp47 is missing from Mtl1 (ref. 41). Our biochemical analysis suggests that the Zn-finger protein, Red1, is responsible for connecting Mtl1 and the MTREC complex to the exosome. This interaction is dependent on Rrp6, a nuclear compartment-specific subunit of the exosome. Consistently, deletion of *red1* or *rrp6* leads to the accumulation of CUTs, meiotic mRNAs and also unspliced pre-mRNA transcripts. This result also highlights the fact that although the Mtl1, Ctr1 and Nrl1 proteins form a biochemically separate complex from MTREC, the role of this complex in splicing quality control is dependent on Red1 and the MTREC complex.

**Figure 5:**
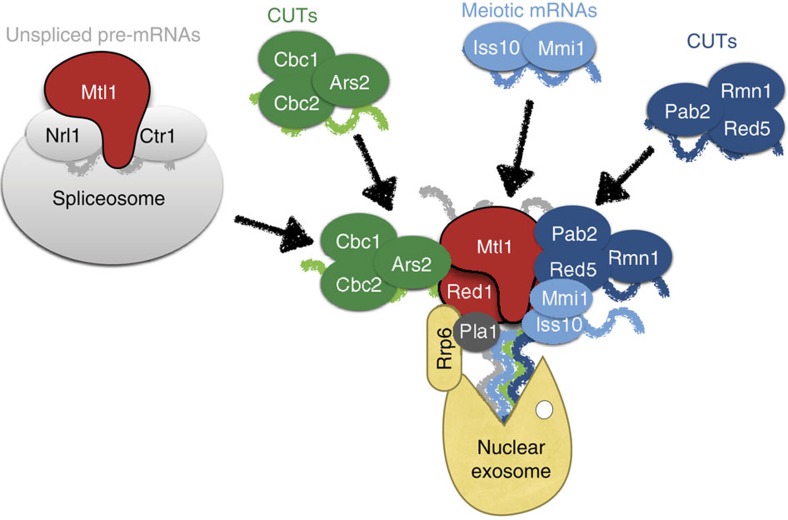
Proposed model for the role of MTREC complex in RNA surveillance. Submodules of the MTREC complex, together with the Mtl1–Ctr1–Nrl1 complex, are recruited to different subsets of CUTs, meiotic mRNAs or unspliced pre-mRNA transcripts and deliver these RNAs to the MTREC complex. The RNAs are polyadenylated by the canonical poly(A) polymerase, Pla1. The RNA-loaded MTREC complex can dock to the nuclear exosome through the Red1–Rrp6 interaction. The helicase activity of the Mtl1 subunit then feeds the MTREC-bound RNAs into the exosome channel.

Nearly all subunits of the MTREC complex contain motifs with potential RNA-binding activity: Cbc1 and Cbc2 bind to the 5′ cap structure of RNA polymerase II transcripts, Rmn1, Pab2 and Mmi1 contain classical RNA-binding motifs, while Red1, Ars2 and Red5 have Zn-finger domains. These subunits might possess different RNA-binding preferences and/or targeting mechanisms and allow the specific recognition of different classes of CUTs and other aberrant transcripts. Indeed, the Mmi1 subunit specifically binds to transcripts with DSR motifs found in a subset of meiotic mRNAs^22^. Consequently, deletion of *mmi1* or its binding partner, *iss10*, leads to the stabilization of meiotic mRNAs, but does not affect the degradation of CUTs. Deletion of *pab2* leads to the strong accumulation of PROMPTs, but only marginally affects the level of AS transcripts. Overall, these results suggest a model in which the MTREC submodules specifically bind, or are recruited to, different subsets of CUTs, unspliced transcripts or meiotic mRNAs, and deliver these RNAs to the MTREC complex. The RNA-loaded MTREC complex then docks to the nuclear exosome through the Red1 subunit and the helicase activity of Mtl1 feeds these RNAs into the exosome channel (Fig. 5).

Our model suggests that the Red1 subunit is indispensable for the nuclear exosome targeting activity of the MTREC complex. Indeed, deletion of *red1* equally affects all subtypes of CUTs, meiotic mRNAs and unspliced pre-mRNAs, and its phenotype is similar to the *rrp6Δ*. Interestingly, neither Red1 nor Rrp6 is essential for cell viability in *S. pombe*, while other subunits, including Mtl1, Red5 and the entire CBCA (Cbc1–Cbc2–Ars2) complex, are essential. This indicates that these subunits must also have additional roles in *S. pombe*. Indeed, Cbc1 and Cbc2 are essential components of the cap structure of mRNAs and Red5 was reported to play a role in mRNA export^27^,^42^. Mtl1 was also shown to be involved in sn(o)RNA processing^29^, like its *S. cerevisiae* homologue, Mtr4.

Remarkably, several evolutionarily conserved subunits of the MTREC complex do not have an apparent homologue in *S. cerevisiae*, including Red1, Red5, Rmn1 and Ars2, indicating that the entire MTREC complex is missing from budding yeast. Consistently, the recognition and exosome targeting of CUTs in *S. cerevisiae* shows major differences to *S. pombe* and to higher eukaryotes. In *S. cerevisiae*, the Nrd1–Nab3 complex recognizes CUTs via sequence-specific RNA–protein interactions and terminates their transcription. These RNA motifs are highly enriched in CUTs and depleted from mRNAs^43^. The TRAMP complex then adds a short oligo(A) tail to these transcripts^7^,^8^,^14^ and targets them to the nuclear exosome. In contrast, the TRAMP complex is mostly dispensable for the degradation of CUTs in *S. pombe* and in higher eukaryotes, and its function is probably more related to nucleolar RNA processing. Similarly, the Nrd1–Nab3 complex is not conserved in higher eukaryotes, and our data shows that deletion of *sen1* in *S. pombe* causes only minor accumulation of CUTs (Supplementary Fig. 2a). The major nuclear exosome targeting factor for CUTs and aberrant transcripts in *S. pombe* and in humans is the MTREC and NEXT complex, respectively. The classical poly(A) cleavage sites are highly enriched upstream of the TSS in these species, suggesting that the canonical cleavage and poly(A) machinery are involved in the processing of CUTs^44^. This is consistent with the finding that CUTs and meiotic mRNAs have long poly(A) tails and the MTREC complex interacts with mRNA cleavage and polyadenylation factors, including the canonical poly(A) polymerase Pla1 (refs 25, 29, 30).

The overlapping functions of the fission yeast MTREC complex and the human NEXT complex raise the question of whether the MTREC complex might be the *S. pombe* orthologue of the NEXT complex. In the *Schizosaccharomyces* genus, the *mtr4* gene went through a duplication event and the two *mtr4* homologues, *mtr4* and *mtl1*, slightly differentiated to fulfil different functions (Supplementary Fig. 5). While Mtr4 is a subunit of the TRAMP complex, Mtl1 is a part of the MTREC complex and the functionally connected Mtl1–Ctr1–Nrl1 complex. Human Mtr4 (hMTR4) is a subunit of both the TRAMP complex and the NEXT complex. However, the additional two subunits of the NEXT complex, the Zn-finger protein ZCCHC8 and the RNA-binding protein RBM7, do not show strong homology with subunits of the MTREC complex. Remarkably, purification of the hMTR4 protein also specifically enriched the predicted human homologues of the MTREC complex, including the Red1 homologue ZFC3H1, the Cbc1 and Cbc2 homologues CBP80 and CBP20, the human ARS2, the Pab2 homologue PABPN1, the Rmn1 homologue RBM27 and the Red5-related Zn-finger protein, ZC3H18 (ref. 17, 18). These data suggest the existence of several different nuclear exosome targeting complexes in higher eukaryotes, including the human TRAMP and NEXT complexes and probably the human orthologue of the MTREC complex. All these complexes share the conserved helicase subunit, hMTR4, but have different Zn-finger- and RNA recognition motif (RRM)-containing subunits, likely targeting specific subclasses of CUTs or other aberrant transcripts to the nuclear exosome. The lack of the MTREC and NEXT complexes in budding yeast might represent another example of the co-elimination of a set of functionally connected genes from *S. cerevisiae*^45^. The Nrd1–TRAMP pathway probably took over the function of these NEXT-like complexes in *S. cerevisiae* to ensure the proper degradation of unwanted ncRNAs in the nucleus and to maintain genomic integrity. Overall, our study reveals the central role of the evolutionarily conserved MTREC complex in RNA quality control, and in the recognition and elimination of aberrant, cryptic transcripts. We are only beginning to understand how the RNA surveillance machinery distinguishes RNAs destined for degradation from mRNAs and stable ncRNAs. However, these findings push MTREC complex to the forefront of this process. More comprehensive characterization of this complex will help to further dissect the molecular mechanisms underlying RNA surveillance in higher eukaryotes.

## Methods

### Tandem affinity purification

Flag-TEV-protein A (FTP)-tagged bait proteins were harvested from 2 l YEA cultures of yeast strains grown to OD_600_ 1.8–2.2. Cell pellets were snap-frozen in liquid nitrogen and ground into powder using the Cryo-mill MM-400 (Retsch). Cells were resuspended in purification buffer (50 mM HEPES, pH 7.0; 100 mM NaCl; 1.5 mM MgCl_2_; 0.15% NP-40), supplemented with 1 mM dithiothreitol (Carl Roth), 1 mM phenylmethylsulphonyl fluoride (Sigma-Aldrich) and protease inhibitor mix (Serva Electrophoresis). Cell extracts were centrifuged at 3,500*g* for 10 min at 4 °C, then the supernatants were further centrifuged at 27,000*g* for 45 min at 4 °C. Clarified supernatants were then incubated with 150 μl slurry of IgG beads (GE Healthcare) for 2 h at 4 °C on a turning wheel. After binding, the beads were washed with 2 × 15 ml purification buffer and TEV cleavage was performed in purification buffer containing 20 units TEV (Life Technologies), 0.5 mM dithiothreitol and 50 units Benzonase (Millipore) for the removal of nucleic acids, for 2 h at 16 °C. The eluate was collected and incubated with 100 μl slurry of anti-Flag beads (Sigma-Aldrich) for 1 h at 4 °C. The protein-bound anti-Flag beads were washed with 2 × 10 ml  purification buffer and the proteins were subsequently eluted from the beads by competition with 200 μl Flag peptide (100 μg ml^−1^, Sigma-Aldrich). The eluted proteins were analysed by Coomassie staining using Brilliant Blue G (Sigma-Aldrich) or silver staining using a SilverXpress staining kit (Life Technologies) on 4–12% NuPAGE Bis-Tris gels (Life Technologies), followed by mass spectrometry analysis.

### Mass spectrometry analysis

Mass spectrometry was performed at the ‘FingerPrints' Proteomics and Mass Spectrometry Facility at the College of Life Sciences, University of Dundee. The raw data were processed with MaxQuant v1.3.0.5 software^46^. The protein search was performed against a curated *S. pombe* protein list. To check the relative protein amounts in the samples, we used the sum of all peptide peak intensities divided by the number of theoretically observable tryptic peptides (iBAQ values)^34^. An identified protein was considered as non-significant if its iBAQ value was <50,000 or had zero spectral counts. iBAQ values were normalized to the iBAQ value of the bait protein and indicated as percentage of the bait. iBAQ values and sequence coverage are shown in Supplementary Data 1 and 2.

### Strand-specific RT–PCR

Total RNAs or RNA isolated from the IP fraction and the input fraction of RIP experiments were treated with Turbo DNase (Life Technologies). RNA concentration was measured using a Qubit RNA Assay Kit (Life Technologies), and 200 ng from the total RNA, or 45 ng from the isolated IP and input fraction of the RIP experiments was reverse transcribed using Superscript III reverse transcriptase (Life Technologies). Gene- and strand-specific primers were used to obtain cDNA. Reverse transcriptions without reverse transcriptase (no RT control) were performed as the negative controls. Primers are listed in the Supplementary Table 2 (* indicates primers used for the reverse transcription step).

### RNA ligation-coupled RT–PCR assay

Turbo DNase-treated total RNAs were incubated with RNase H (New England Biolabs) in the presence or absence of oligo(dT)_12–18_. The resulting RNAs were ligated with a 3′ linker, containing a blocked 3′ (ddC) end and an activated adenosine at the 5′ end using truncated T4 RNA ligase 2 (New England Biolabs). RNAs were then purified using a NucleoSpin miRNA kit (Macherey-Nagel) to remove free 3′ linker oligos, followed by reverse transcription using RT-primer that anneals to the 3′ linker sequence. cDNAs were purified with Agencourt AMPure XP beads (Beckman Coulter). The first PCR step was performed with only a gene-specific sense primer for 30 cycles, second PCR amplification was performed with nested sense primer labelled at the 5′ end with [γ-^32^P]ATP using T4 polynucleotide kinase (New England Biolabs) and reverse primer annealing to the 3′ linker. Primers are listed in the Supplementary Table 2 (^#^ indicates primers labelled with ^32^P). The resulting PCR products, as well as a 50-bp DNA ladder (Life Technologies) labelled with [γ-^32^P]ATP, were loaded on 6% acrylamide gels (SequaGel UreaGel System, National Diagnostics). The gel was then placed on a piece of Whatman filter paper, dried under vacuum and heat (80 °C), exposed and scanned on a Fujifilm Image Reader (Fujifilm FLA 7000).

### Expression arrays

WT and mutant strains were grown in YEA at 30 °C to OD_600_ 0.5–0.8. Temperature-sensitive strains were grown in YEA at 23 °C to OD_600_ 0.3–0.4 and then moved to 37 °C for 4 h (OD_600_<0.8). Total RNAs from WT and mutants were isolated using the TRI Reagent (Sigma Aldrich), followed by rRNA depletion and reverse transcription using the SuperScript Indirect cDNA labelling system (Life Technologies) with anchored oligo(dT)_20_ and random hexamers. cDNAs from WT and mutant strains were labelled with Cy3 and Cy5 (GE Healthcare), respectively, and hybridized to high-resolution tiling microarrays (Agilent) consisting of forward- and reverse-DNA-strand-specific probes. Expression arrays were performed with at least two biological replicates, except *mmi1Δ* and *rmn1Δ*.

### RNA immunoprecipitation microarray profiling (RIP-Chip)

Tandem affinity purifications of FTP-tagged strains were performed as described above with minor modifications. Reagents were prepared under RNase-free conditions and RNase inhibitor (Fermentas) was used during purification. IPed RNA–protein complexes were eluted from anti-Flag beads, followed by the phenol/chloroform/isoamyl alcohol extraction of RNAs. Total RNAs from the input were extracted using the TRI Reagent (Sigma Aldrich). The IPed RNAs and input RNAs were then treated using Turbo DNase (Life Technologies) to remove the potential DNA contamination. Reverse transcription was performed using the SuperScript Indirect cDNA labelling system (Life Technologies) with random hexamers. The cDNAs from IPed and input samples were labelled with Cy5 and Cy3 (GE Healthcare), respectively, and hybridized to high-resolution tiling microarrays (Agilent). RIP-Chip experiments were performed with at least two biological replicates.

### Microarray design

Expression arrays and RIP-Chip arrays were hybridized to Agilent custom microarray design with 180-K probes (Design ID:036227). The microarrays cover ∼50% of the *S. pombe* genome (chr2, chr3:1-132300; 2,320 genes) with 40-bp resolution, and the remaining 50% with 250-bp resolution. Probes alternate on the forward and reverse strand. Probe length is between 30 and 60 nt Tm optimized (75 °C). This array design allows us to monitor gene expression changes (sense, AS and intergenic) in the entire genome, and to obtain a more detailed high-resolution analysis for half of the *S. pombe* genome. Scanning and initial processing of microarrays were performed using Agilent DNA Microarray Scanner and Agilent Scan Control software (version A.8.4.1.). Data extraction was performed using Agilent feature extraction software (version 10.7.3.1.).

### RNA sequencing

rRNAs were depleted from Turbo DNase-treated total RNAs using a Ribo-zero Magnetic Gold Kit (Epicentre). The cDNA libraries were prepared using a NEBNext Ultra RNA Directional Library Prep Kit (New England Biolabs) according to the manufacturer's instructions. The libraries were purified using Agencourt AMPure XP beads (Beckman Coulter) and assessed on a Bioanalyzer (Agilent). Subsequently, 100 bp paired-end sequencing was performed on an Illumina HiSeq 2000 sequencer at BGI Tech (Hong Kong). RNA-seq data were analysed using TopHat 2.0.13 (Version 2.2.2)^47^, SAMtools^48^ and R-Bioconductor (2.14) ShortRead packages. The median RNA-seq read coverage of intronless genes was normalized to 100 in all datasets.

### Composite profiles of expression arrays and RIP experiments

Composite profiles were generated with the help of GenomicRanges (1.16.3) R-package using the *S. pombe* 2007_April gene annotation. For the sense analysis, we used genes represented on the high-resolution part of our array and had at least 600-bp intergenic region in the 3′ direction and 100-bp intergenic region in the 5′ direction (993 genes). For the AS analysis, we used genes represented on the high-resolution part of our array and had at least 600-bp intergenic region in the 5′ direction and at least 100 bp in the 3′ direction (1,569 genes). Genes used in the analyses were divided into 15 equal parts (bins), the mean ratio (mutant/WT or RNA-IP/input) of the sense or AS probes were determined for each bin. For AS or the sense analysis, 5′ or 3′ regions (10–10 probes, equivalent of 800–800 bp) were also included, respectively. In the resulting matrix, each row represents a gene and each column represents the position in the gene region (flanking regions and bins 1–15). The average of the log_2_ values (geometric average) was determined for each column and plotted on a log_2_ scale separately for sense (upper plot) and AS (lower plot with mirrored axis) transcripts. Only positive values are shown on the plots. The mean sense gene expression was normalized to WT (0 on the log_2_ scale) to compensate the artificial differences caused by the increased AS and intergenic RNA transcripts in the mutant strains.

### Composite profiles of RNA-seq data on intronic sequences

Composite profiles were generated with the help of GenomicRanges (1.16.3) R-package. In Supplementary Fig. 2b, we used *S. pombe* 2007_April annotation of mRNAs and excluded genes with the highest and lowest 10% expression levels in the WT strain (4,041 genes used). For the analysis in Fig. 4b and Supplementary Fig. 4a, we used *S. pombe* Ensembl EF2 intron annotation, only introns in open reading frames (ORFs) and between 20 and 400 bp in length, excluded 10–10% of the introns with the lowest and highest expression levels of the corresponding genes (estimated from the median RNA-seq read coverage of the surrounding 20–20 bp exons in WT strain (3,935 introns used)). For the analysis in Supplementary Fig. 4d, we used *S. pombe* Ensembl EF2 intron annotation, only introns in ORFs and between 20 and 400 bp in length and excluded introns with the highest and lowest 10% expression levels of the corresponding genes (estimated from the median RNA-seq read coverage of the surrounding 20–20 bp exons in WT strain). Introns were ranked by the change in the average RNA-seq read coverage in the *ctr1Δ* strain compared with the WT, normalized by the expression level of the surrounding exons in the *ctr1Δ* strain. Introns in the upper 25 percentile were used for the analysis to select for the largest increase in intronic coverage relative to the gene expression (984 introns used). In Supplementary Fig. 4c, we used *S. pombe* Ensembl EF2 annotation of intronless ORFs, and excluded genes with the highest and lowest 10% expression levels in the WT strain (1,886 ORFs used).

### Phylogenetic analysis

The homologues for the *S. pombe* proteins, SPCC550.03c (Ski2), SPAC6F12.16c (Mtr4) and SPAC17H9.02 (Mtl1), were searched for in the National Center for Biotechnology Information database with BLAST, in *D. melanogaster*, *H. sapiens*, *N. crassa*, *S. cerevisiae*, *S. japonicus* and *S. pombe*. The hits were fed into the phylogeny program at phylogeny.fr^49^, using T-Coffee alignment^50^, Gblocks curation^51^ and PhyML phylogeny^52^, using aLRT^53^ test for branch support.

## Additional Information

**Accession codes:** Genomic data have been deposited in NCBI's Gene Expression Omnibus^54^ and are accessible through GEO Series accession number GSE64992. The mass spectrometry proteomics data have been deposited at the ProteomeXchange Consortium^55^ via the PRIDE partner repository with the dataset identifier PXD001908.

**How to cite this article**: Zhou, Y. *et al*. The fission yeast MTREC complex targets CUTs and unspliced pre-mRNAs to the nuclear exosome. *Nat. Commun.* 6:7050 doi: 10.1038/ncomms8050 (2015).

## Supplementary Material

Supplementary Figures, Supplementary Tables and Supplementary References Supplementary Figures 1-5, Supplementary Tables 1-2 and Supplementary References

Supplementary Data 1 Intensity-based absolute quantification (iBAQ) of the purified proteins. The table summarizes the results of the LC-MS/MS analysis of the purified total protein fractions showing the iBAQ of the identified proteins. The abundance of the indicated proteins was color-coded, as in Supplementary Figure 1. (Black: = 2.5% of the bait, Gray: = 0.5% but < 2.5% of the bait, White: non-significant or < 0.5% of the bait). Colored borders indicate the different submodules of the MTREC complex, as identified in the analysis.

Supplementary Data 2 Percentage sequence coverage of the purified proteins. The table summarizes the results of the LC-MS/MS analysis of the purified total protein fractions showing the percentage sequence coverage of the identified proteins. The abundance of the indicated proteins was color-coded, as in Supplementary Figure 1. (Black: = 2.5% of the bait, Gray: = 0.5% but < 2.5% of the bait, White: non-significant or < 0.5% of the bait). Colored borders indicate the different submodules of the MTREC complex, as identified in the analysis.

## Figures and Tables

**Table 1:** Summary of the extended biochemical analysis of the MTREC complex.

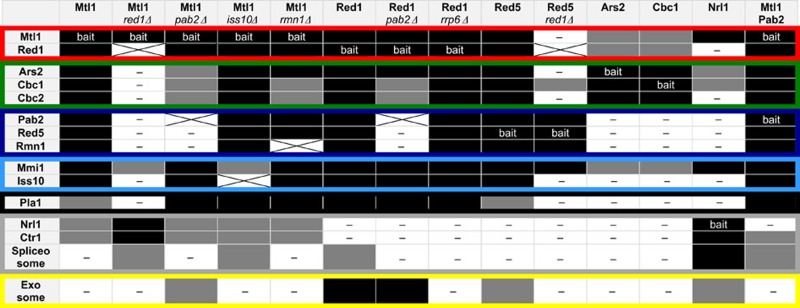 [Fn t1-fn1]

The top row shows the purified bait proteins (note that the last column represents split-tag purification with two bait proteins) and, where the strain used was not WT, the genetic background of the strain used for the purification is indicated in italics. The abundance of the indicated proteins were colour-coded as follows: black: ⩾2.5% of the bait, grey: ⩾0.5% but <2.5% of the bait, white(—): non-significant or <0.5% of the bait; coloured borders indicate the different submodules of the MTREC complex, as identified in the analysis (see also Fig. 1b). The abundance of the ‘Exosome' includes the average abundance of all 12 nuclear exosome subunits. The abundance of the ‘Spliceosome' includes the average abundance of the subunits of the Prp19 complex.
