# Supplementary material for: The fission yeast MTREC complex targets CUTs and unspliced pre-mRNAs to the nuclear exosome
Source: Nat Commun. 2015 May 20;6:7050. doi: 10.1038/ncomms8050 (PMC4455066; doi:10.1038/ncomms8050)
Supplement: Supplementary Figures, Supplementary Tables and Supplementary References — Supplementary Figures 1-5, Supplementary Tables 1-2 and Supplementary References [file ncomms8050-s1.pdf]

Supplementary Figure 1

a)

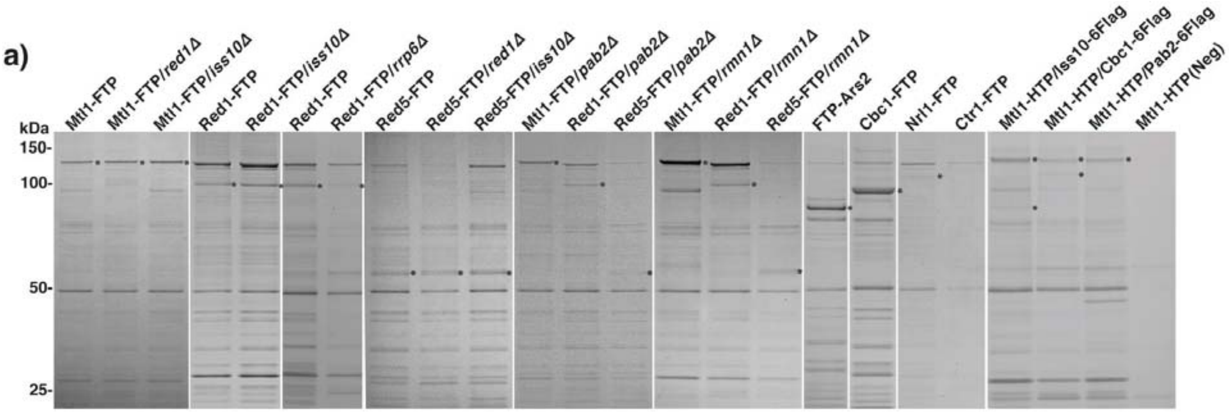

b)

	Mtl1	Mtl1 red1Δ	Mtl1 pab2Δ	Mtl1 iss10Δ	Mtl1 rmn1Δ	Red1	Red1 pab2Δ	Red1 rrp6Δ	Red1 iss10Δ	Red1 rmn1Δ	Red5
Mtl1	100.00	100.00	100.00	100.00	100.00	116.98	128.24	127.93	159.44	144.16	25.95
Red1	70.77		54.86	69.28	69.00	100.00	100.00	100.00	100.00	100.00	25.99
Ars2	11.18	-	1.42	10.62	5.92	8.53	3.21	13.93	6.76	4.00	5.84
Cbc1	5.52	-	0.69	5.52	2.49	2.99	1.00	5.84	2.34	0.85	3.56
Cbc2	6.69	0.40	0.59	5.06	2.31	3.32	1.89	8.10	2.54	1.21	3.92
Pab2	12.97	-		17.20	2.91	2.72		29.36	1.05	1.78	17.64
Red5	8.75	-	-	6.06	2.56	2.58	-	5.29	2.59	1.08	100.00
Rmn1	21.52	-	-	14.51		6.15	-	30.07	3.99		21.99
Mmi1	4.45	0.87	4.02	1.12	5.22	50.39	22.02	16.81	2.28	17.88	7.04
Iss10	15.34	-	10.99		13.23	16.69	13.04	21.39		20.49	6.16
Pla1	1.90	0.22	3.11	3.91	3.13	3.70	5.76	6.74	1.66	2.45	1.79
Nrl1	1.56	12.95	1.76	2.28	1.51	-	-	-	-	-	-
Ctr1	1.79	14.78	1.00	2.40	1.54	-	-	-	-	-	-
Spliceosome	0.23	1.34	0.40	0.52	0.26	0.57	0.46	0.21	0.32	0.25	0.30
Exosome	0.10	0.12	0.66	0.43	0.35	22.49	21.83	0.16	20.84	17.52	0.73

	Red5 red1Δ	Red5 pab2Δ	Red5 iss10Δ	Red5 rmn1Δ	Ars2	Cbc1	Nrl1	Ctr1	Mtl1 Iss10	Mtl1 Cbc1	Mtl1 Pab2	Mtl1 (Neg)
Mtl1	-	-	26.49	6.68	2.04	1.58	192.98	219.43	100.00	100.00	100.00	1.39
Red1		1.22	28.13	10.26	2.35	1.98	-	-	113.43	106.79	118.44	1.65
Ars2	-	-	5.28	2.56	100.00	14.77	0.98	-	19.33	47.85	22.77	-
Cbc1	0.74	-	2.75	0.96	10.30	100.00	1.03	-	8.85	56.36	8.68	-
Cbc2	-	-	1.90	1.31	20.62	148.17	-	-	10.77	58.72	22.64	-
Pab2	10.11		17.74	7.36	-	-	-	-	11.56	14.90	246.27	-
Red5	100.00	100.00	100.00	100.00	-	-	-	-	8.35	9.43	34.65	-
Rmn1	12.39	-	22.36		0.18	0.23	-	-	25.67	51.37	55.77	-
Mmi1	6.51	2.61	2.77	5.43	1.55	1.24	6.92	-	17.86	83.13	34.31	-
Iss10	-	-		3.13	0.47	0.38	-	-	67.06	27.01	29.93	-
Pla1	-	1.66	1.21	0.75	-	0.27	-	-	5.99	8.88	9.23	-
Nrl1	-	-	-	-	-	-	100.00	19.57	0.69	-	-	-
Ctr1	-	-	-	-	-	-	94.25	100.00	1.64	-	1.92	-
Spliceosome	0.33	0.41	0.22	0.22	0.09	0.47	16.14	47.32	0.35	1.02	0.75	0.01
Exosome	0.33	0.53	1.14	0.22	0.03	0.27	0.82	0.93	0.63	0.66	0.44	0.00

Complete set of the biochemical analysis of the MTREC complex.

a) Coomassie blue stained or silver stained (Red1-FTP, Red1-FTP/*rrp6*Δ) SDS polyacrylamide gels show the indicated tandem affinity-purified proteins in the presence of Benzonase. The genetic background of the strains is indicated, unless the WT strain was used. The bait proteins are indicated by *. The last gel panel on the right shows the results of the split-tag purifications using Mtl1-HTP as the first purification step and a Flag purification of the indicated 6Flag-tagged protein as the second purification step. The Mtl1-HTP(Neg) served as a negative control without any Flag-tagged proteins in the strain. b) The summary of the LC-MS/MS analysis of the purified total protein fractions showing the abundance of the identified proteins normalized to the bait, as a percentage. In the split-tag purifications, the abundance was normalized to Mtl1. In the Mtl1(Neg) negative control, the abundance was normalized to the amount of Mtl1 in the Mtl1-Pab2 purification. The abundance of the indicated proteins were color-coded as follows: Black: ≥ 2.5% of the bait, Gray: ≥ 0.5% but < 2.5% of the bait, White: < 0.5% of the bait, (-): non-significant; Colored borders indicate the different submodules of the MTREC complex, as identified in the analysis. The abundance of the 'Exosome' includes the average abundance of all 12 nuclear exosome subunits. The abundance of the 'Spliceosome' includes the average abundance of the subunits of the Prp19 complex. A subset of this analysis was presented in Table 1.

Supplementary Figure 2

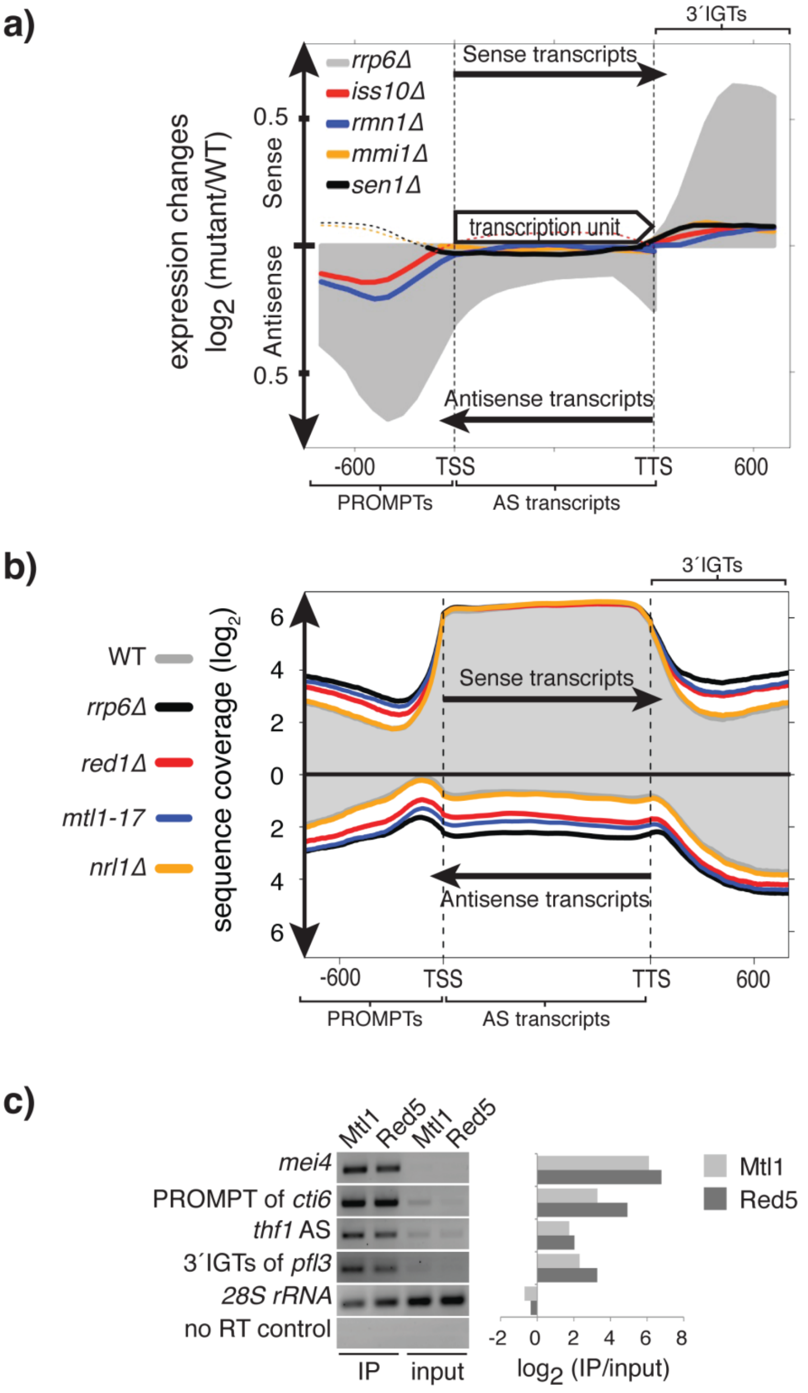

MTREC complex is the major nuclear exosome targeting factor in *S. pombe*.

a) Composite plot of relative RNA levels in the indicated mutant strains, compared to WT was generated as described in Figure 2 b. The gray shaded area represents the average expression changes in the *rrp6*Δ strain and colored lines in the indicated strains. Dotted lines represent negative values (RNA amount is decreased compared to WT). b) Composite plot of RNA-seq read coverage of protein-coding genes in the *S. pombe* genome. Each gene was divided into 120 parts (bins) and the average RNA-seq coverage in the S and AS direction was determined for each bin. In addition, 800 bp from the flanking 5′ and 3′ regions were included in the analysis. In the resulting matrix, each row represents a gene and each column represents the position in the gene region (flanking regions and bins 1-120). The average of the $\log_2$ values (geometric average) was determined for each column and plotted on a $\log_2$ scale separately for S (upper plot) and AS (lower plot with mirrored axis) transcripts. The gray shaded area represents the average RNA level in the WT strain and colored lines in the indicated strains. c) Strand-specific RT-PCR of Mtl1-FTP and Red5-FTP RIP experiments. The *mei4* meiotic mRNA, 28S rRNA and selected CUTs (including PROMPT of *cti6*, *thf1* AS and 3′IGT of *pfl3*) were amplified in a strand-specific RT-PCR in the IP fraction and in the input. A no RT reaction for *thf1* AS was performed to exclude the presence of DNA contamination. Right panel shows the quantification of the enrichment ($\log_2$ scale) of the corresponding RNA in the IP fraction compared to input.

Supplementary Figure 3

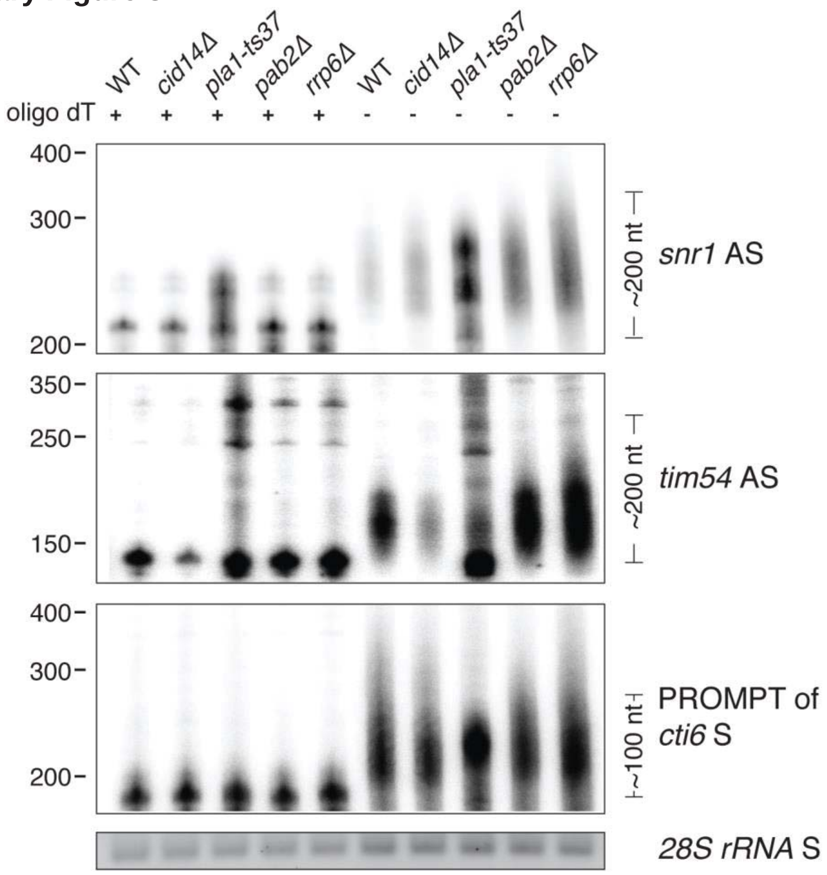

CUTs and meiotic mRNAs have long poly(A) tails.
RNA ligation-coupled RT-PCR assay to assess the poly(A) tail length of the indicated transcripts in WT and mutant cells. See Figure 3 for details.

Supplementary Figure 4

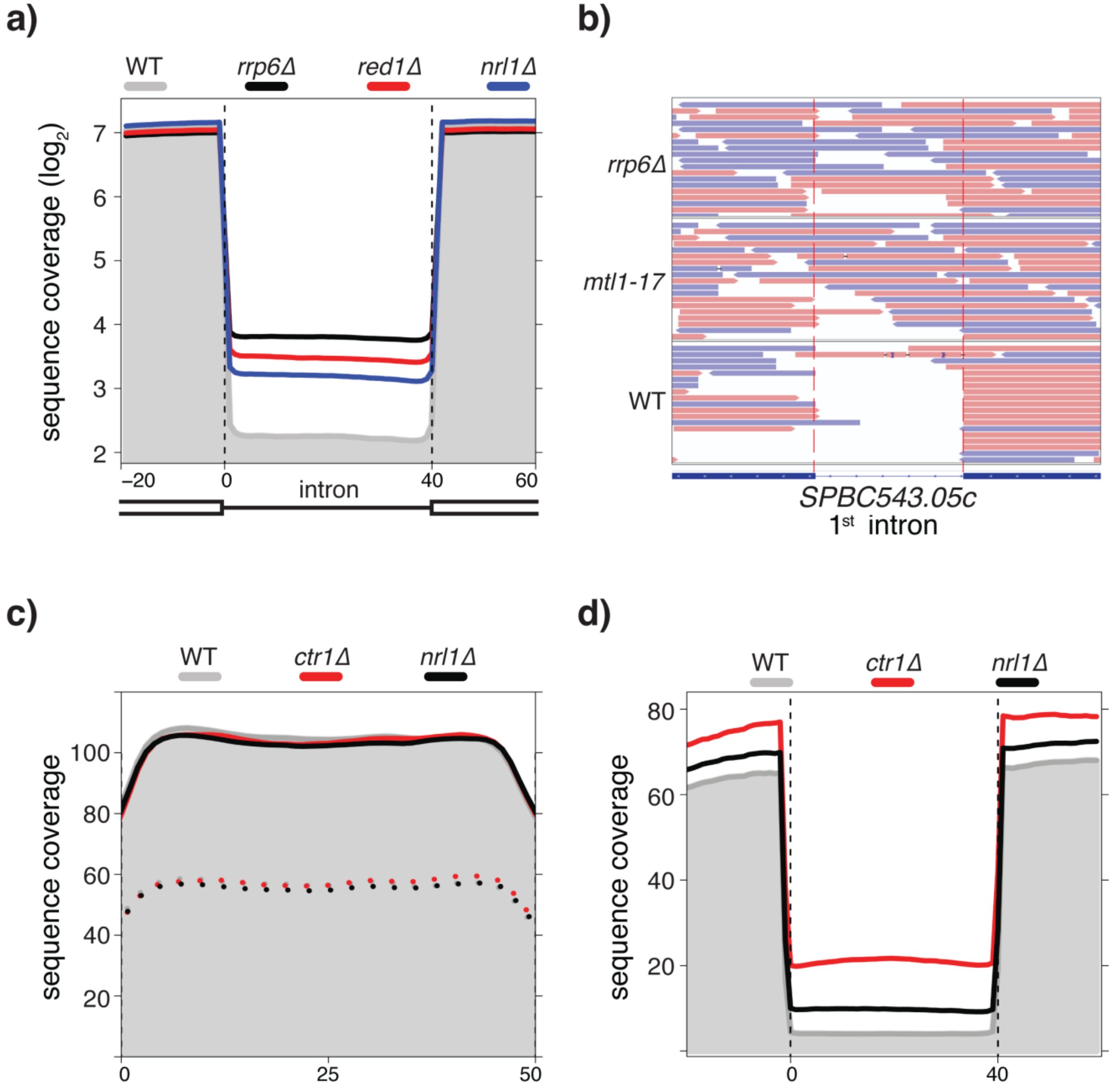

The Mtl1-Ctr1-Nrl1 complex targets unspliced RNA products to the MTREC complex.

a) Composite plot of RNA-seq read coverage, including all introns in genes with detectable expression (3935 introns), was generated as described in Figure 4 b. The gray shaded area represents the average RNA levels in the WT strain and colored lines in the indicated mutant strains. b) Strand-specific RNA-seq reads of a representative exon-intron junction (*SPBC543.05c* 1st intron), showing 100 bp paired-end Illumina sequence reads aligned to the genome in WT and indicated mutant strains. Blue and red lines represent the 1st and 2nd pair reads, respectively. c) Composite plot of RNA-seq read coverage including intronless genes in the *S. pombe* genome. Each gene was divided into 50 parts (bins) and the average RNA-seq read coverage (only S transcripts) was determined for each bin. In the resulting matrix, each row represents an intronless gene and each column represents the position in the gene (bins 1-50). The average of the log_2 values (geometric average) was determined for each column and plotted on a linear scale. The gray shaded area represents the average RNA levels in the WT strain and colored lines in the indicated mutant strains. The dotted lines represent the same analysis carried out with the genes in the lower 50 percentile of expression levels (943 genes used). d) Composite plot of RNA-seq read coverage including a subset of introns in the *S. pombe* genome. Introns were ranked by the change in average RNA-seq read coverage in the *ctr1*Δ strain compared to the WT normalized by the expression level of the surrounding exons in the *ctr1*Δ strain. Introns in the upper 25 percentile were used for the analysis in order to select for the largest increase in intronic coverage relative to the gene expression (984 introns used). The analysis was performed as described in Figure 4 b, with the exception that the values were plotted on a linear scale. The gray shaded area represents the average RNA levels in the WT strain and colored lines in the indicated mutant strains.

Supplementary Figure 5

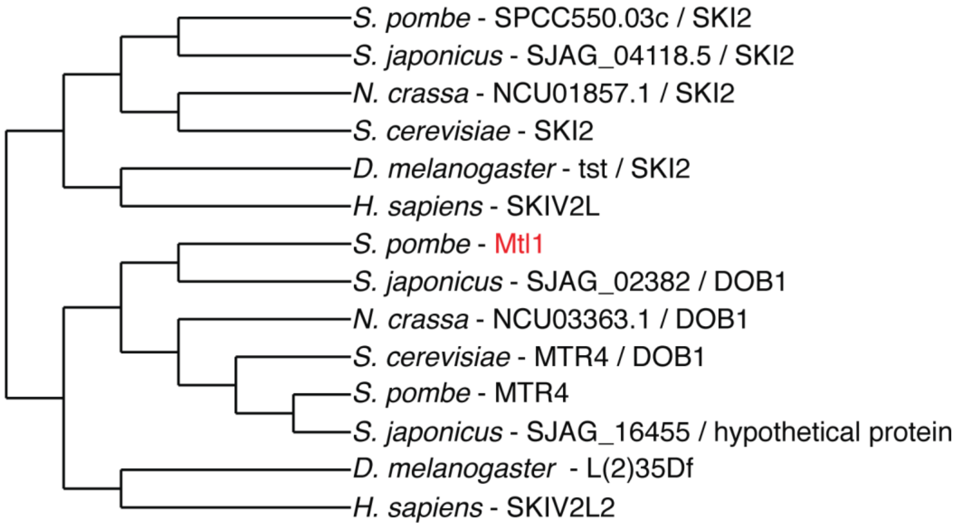

Phylogenetic tree of Mtr4 homologs.
Cladogram of the Mtr4-like proteins from the species *Schizosaccharomyces pombe*, *Schizosaccharomyces japonicus*, *Neurospora crassa*, *Saccharomyces cerevisiae*, *Drosophila melanogaster* and *Homo sapiens*. The upper main cluster contains the Ski2 homologs while the lower part contains the Mtr4 homologs. All members of the *Schizosaccharomyces* genus have 2 homologs of Mtr4 (*Schizosaccharomyces octosporus* and *Schizosaccharomyces cryophylus* not shown).

Supplementary Table 1

Yeast strains used in this study

Strain	Genotype	Source
P20(WT)	*h+, leu1-32, ura4 DS/E, ade6-216, otr1R(SphI)::ura4+*	this study
P344(WT)	*h+, leu1-32, ura4D18, ade6-M210*	V2-33-H11, Bioneer Inc
P345(WT)	*h+, leu1-32, ura4D18, ade6-216*	V2-33-H12, Bioneer Inc
P918(WT)	*h90, leu1-32, ura4D18, ade6-M210*	Sugiyama et al (2011)[1]
P74(*rrp6Δ*)	*h+, leu1-32, ura4, ade6-216, rrp6Δ::kanMX6*	this study
P919(Red1-FTP)	*h90, leu1-32, ura4D18, ade6-M210, red1-FTP::natNT2*	this study
P920(Mtl1-FTP)	*h90, leu1-32, ura4D18, ade6-M210, mtl1-FTP::natNT2*	this study
P931(Mtl1-Flag)	*h90, leu1-32, ura4D18, ade6-M210, mtl1-3xFlag::kanMX6*	this study
P934(*red1Δ*)	*h90, leu1-32, ura4D18, ade6-M210, red1Δ::kanMX6*	Sugiyama et al (2011)[1]
P935(*iss10Δ*)	*h90, leu1-32, ura4D18, ade6-M210, iss10Δ::kanMX6*	this study
P741(*pab2Δ*)	*h+, leu1-32, ade6-216, ura4D18, pab2Δ::kanMX6*	V2-17-G06, Bioneer Inc
P936(*mtl1-17-Flag*)	*h90, leu1-32, ura4D18, ade6-M210, mtl1-17-3xFlag::kanMX6*	this study
P937(*red5-2-Flag*)	*h90, leu1-32, ura4D18, ade6-M210, red5-2-3xFlag::kanMX6*	Sugiyama et al (2013)[2]
P938(*cid14Δ*)	*h90, leu1-32, ura4D18, ade6-M210, cid14Δ::kanMX6*	this study
P850(*sen1Δ*)	*h+, leu1-32, ura4D18, ade6-M210, sen1Δ::kanMX6*	V2-12-C05, Bioneer Inc
P962(*rmn1Δ*)	*h90, leu1-32, ura4D18, ade6-M210, rmn1Δ::kanMX6*	this study
P983(*mei4-P572*)	*h-, leu1, mei4-P572*	Sugiyama et al (2011)[1]
P984(*mmi1Δ*)	*h-, leu1, mei4-P572, mmi1Δ::kanMX6*	Sugiyama et al (2011)[1]
P1062(Red1-FTP *iss10Δ*)	*h90, leu1-32, ura4D18, ade6-M210, red1-FTP::natNT2, iss10Δ::kanMX6*	this study
P1064(Mtl1-FTP *red1Δ*)	*h90, leu1-32, ura4D18, ade6-M210, mtl1-FTP::natNT2, red1Δ::kanMX6*	this study
P1066(Mtl1-FTP *iss10Δ*)	*h90, leu1-32, ura4D18, ade6-M210, mtl1-FTP::natNT2, iss10Δ::kanMX6*	this study
P1135(*ctr1Δ*)	*h+, leu1-32, ura4D18, ade6-M210, ctr1Δ::kanMX6*	V2-28-H08, Bioneer Inc
P1160(Red5-FTP)	*h+, leu1-32, ura4D18, ade6-216, red5-FTP*	this study
P1162(Nrl1-FTP)	*h90, leu1-32, ura4D18, ade6-M210, nrl1-FTP::natNT2*	this study
P1163(Ctr1-FTP)	*h90, leu1-32, ura4D18, ade6-M210, ctr1-FTP::natNT2*	this study
P1196(Red5-FTP *red1Δ*)	*h+, leu1-32, ura4D18, ade6-216, red5-FTP, red1Δ::kanMX6*	this study
P1199(Red5-FTP *iss10Δ*)	*h+, leu1-32, ura4D18, ade6-216, red5-FTP, iss10Δ::kanMX6*	this study
P1234(Red1-FTP *rrp6Δ*)	*h90, leu1-32, ura4D18, ade6-M210, red1-FTP::natNT2, rrp6Δ::kanMX6*	this study
P1268(Cbc1-FTP)	*h90, leu1-32, ura4D18, ade6-M210, cbc1-FTP::natNT2*	this study
P1312(Red1-FTP *pab2Δ*)	*h90, leu1-32, ura4D18, ade6-M210, red1-FTP:: natNT2, pab2Δ::kanMX6*	this study

P1316(Mtl1-FTP *pab2∆*)	*h90, leu1-32, ura4D18, ade6-M210, mtl1-FTP:: natNT2, pab2∆::kanMX6*	this study
P1319(Red5- FTP *pab2∆*)	*h+, leu1-32, ura4D18, ade6-216, red5-FTP, pab2∆::kanMX6*	this study
P1351(Red1-FTP *rmn1∆*)	*h90, leu1-32, ura4D18, ade6-M210, red1-FTP:: natNT2, rmn1∆::kanMX6*	this study
P1355(Mtl1-FTP *rmn1∆*)	*h90, leu1-32, ura4D18, ade6-M210, mtl1-FTP:: natNT2, rmn1∆::kanMX6*	this study
P1356(Red5-FTP *rmn1∆*)	*h+, leu1-32, ura4D18, ade6-216, red5-FTP, rmn1∆::kanMX6*	this study
P1365(*nrl1∆*)	*h90, leu1-32, ura4D18, ade6-M210, nrl1∆::kanMX6*	this study
P1429(FTP-Ars2)	*h90, leu1-32, ura4D18, ade6-M210, FTP-ars2(N-terminal)*	this study
P1598(*nrl1∆ ctr1∆*)	*h90, leu1-32, ura4D18, ade6-M210, nrl1∆::kanMX6, ctr1∆::hphNT1*	this study
JT452(*plat1-ts37*)	*h90, leu1, ade6-216, pla1-37-GFP::kan*	Yamanaka et al (2010) [3]
P1085(Mtl1-HTP)	*h+, leu1-32, ura4D18, ade6-216, mtl1-HTP*	this study
P1695(Mtl1-HTP Pab2-6xFlag)	*h+, leu1-32, ura4D18, ade6-216, mtl1-HTP, pab2-6xFlag::kanMX6*	this study
P1810(Mtl1-HTP Iss10-6xFlag)	*h+, leu1-32, ura4D18, ade6-216, mtl1-HTP, iss10-6xFlag::kanMX6*	this study
P1848(Mtl1-HTP Cbc1-6xFlag)	*h+, leu1-32 ura4D18, ade6-216, mtl1-HTP, cbc1-6xFlag::kanMX6*	this study

Supplementary Table 2

Primers used in this study

Name	Forward primer	Reverse primer
**Primers used for RIP-RT-PCR**		
mei4	TAAACCCCCTCTTCCCCCTT	ACATCCTCTTCTTCATTGGCAG*
PROMPT of *cti6*	CCTTGACCATCGGAGAACCT	AGCAGCAGCACCTTACAGAAAT*
thf1 AS	GTTGACGAACACATGCAATAGC	CTTGGCTTCCGAAATTGGTATC*
3´IGTs of *pfl3*	TTACCGATGATTCCAGTAGC	AACCTTGCCAGTGTTCTATG*
28S rRNA	ATAACGGCTGAACGCCTCTAAG	CGCCCATCTTCTACGATACAG*
**Primers used for RT-PCR to detect US and S RNAs**		
SPBC119.18	ATGTCGTCCTCTGTTAGTGAAG	TTCAAAGCCTTCAAAAGACAT*
psy2	GAGATAGTTTGGTTCGTTATGTTTC	GAGAACAACGATTCGAGTAAAGG*
SPCC132.03	CCTGAGGATTTGACTGATGCTTAC	CACAGATCGTAGAACTGGCTCAG*
28S rRNA	ATAACGGCTGAACGCCTCTAAG	CGCCCATCTTCTACGATACAG*
GAPDH	AACATCATCCCCTCCTCCAC	GCCTTGATGTCCTCGTAGTTG*
**Primers used for RNA ligation coupled RT-PCR assay**		
3´ linker	/5rApp/CTGCTGCTAGCCTAGGCTA/3ddC/	
RT_polyA	GGTAAGCAGATCAGATCGAAGTCTCCTA GCCTAGGCTAGCAGCAG	
polyA_r	TCTCCTAGCCTAGGCTAGCAGCAG	
*snr1*_AS_f1	ATTAGCTGGGCAAATACTTGTCTC	
*snr1*_AS_f2	TTTAGTTTCTCAGGGTTTGTCG#	
*tim54*_AS_f1	TATCTATCGGTTTCTCCCTCTTT	
*tim54*_AS_f2	CGTTTATTTGCGAATCAATATATGG#	
PROMPT of *cti6*_f1	GCAATCTTTAGAATGGCGTATGT	
PROMPT of *cti6*_f2	AAGGCTTTTTCCTCGCAATGTC#	
*rec8*_f1	TTACAACTCTCTGATGACTTCGTAC	
*rec8*_f2	AAAAGCACCGATGCCATTTG#	
*28S rRNA*_f1	ATAACGGCTGAACGCCTCTAAG	
*28S rRNA*_f2	TTCCTACTCTCCTGTATCGTAGAAG#	

Supplementary References

1. Sugiyama, T. & Sugioka-Sugiyama, R. Red1 promotes the elimination of meiosis-specific mRNAs in vegetatively growing fission yeast. *EMBO J* **30,** 1027-1039 (2011).
2. Sugiyama, T., Wanatabe, N., Kitahata, E., Tani, T. & Sugioka-Sugiyama, R. Red5 and three nuclear pore components are essential for efficient suppression of specific mRNAs during vegetative growth of fission yeast. *Nucleic Acids Res* **41,** 6674-6686 (2013).
3. Yamanaka, S., Yamashita, A., Harigaya, Y., Iwata, R. & Yamamoto, M. Importance of polyadenylation in the selective elimination of meiotic mRNAs in growing S. pombe cells. *EMBO J* **29,** 2173-2181 (2010).
